# Supplementary material for: Natural mismatch repair mutations mediate phenotypic diversity and drug resistance in Cryptococcus deuterogattii
Source: eLife. 2017 Sep 26;6:e28802. doi: 10.7554/eLife.28802 (PMC5614558; doi:10.7554/eLife.28802)
Supplement: Figure 1—source data 1. — Mutations identified on the branch separating VGIIa-like from VGIIa strains with predicted effects and functional domain information about genes affected by the changes. [file elife-28802-fig1-data1.docx]

Figure 1- Source Data 1. Mutations shared by VGIIa-like strains relative to VGIIa

| Mutation | Location | Predicted Effect | Gene | Predicted Function |
| --- | --- | --- | --- | --- |
| SNP | SC2.1:1493731 | Missense (Ser->Pro) | CNBG_0538 | t-RNA ligase |
| SNP | SC2.2:591810 | Splice site acceptor | CNBG_0776 | HAD hydrolase |
| SNP | SC2.2:965997 | Missense (Gly->Asp) | CNBG_0907 | Clp protease subunit |
| SNP | SC2.4:899106 | Missense (Leu->Val) | CNBG_1894 | RhoGEF |
| SNP | SC2.6:160627 | Missense (Ala->Val) | CNBG_2540 | Ubiquitin-like |
| SNP | SC2.6:410824 | Intergenic | - |  |
| SNP | SC2.6:1157157 | Intergenic | - |  |
| SNP | SC2.7:290975 | Missense (His->Tyr) | CNBG_3034 | BRCT domain-containing |
| SNP | SC2.8:594146 | Synonymous | CNBG_3547 | snRNP |
| SNP | SC2.10:94373 | Intronic | CNBG_3750 | Dcp2 |
| SNP | SC2.13:413395 | Missense (Leu->Ser) | CNBG_4876 | esterase |
| SNP | SC2.15:96883 | Intergenic | - |  |
| SNP | SC2.16:143098 | Missense (Pro->Leu) | CNBG_5437 | Protein kinase Nrc2 |
| INDEL | SC2.1:15401-15402 | Intronic | CNBG_0005 | sugar metabolism |
| INDEL | SC2.3:240763-240764 | Nonsense | CNBG_1661 | Msh2 |
| INDEL | SC2.9:241948-241949 | Intronic | CNBG_4065 | SAM methyltransferase |
| INDEL | SC2.11:510573-510575 | Intergenic | - |  |
| INDEL | SC2.14:162875-162876 | Intergenic | - |  |
| INDEL | SC2.15:299154-299155 | Intergenic | - |  |
