# Supplementary material for: Natural mismatch repair mutations mediate phenotypic diversity and drug resistance in Cryptococcus deuterogattii
Source: eLife. 2017 Sep 26;6:e28802. doi: 10.7554/eLife.28802 (PMC5614558; doi:10.7554/eLife.28802)
Supplement: Figure 5—source data 1. — Predicted aneuploid scaffolds for 14 spore progeny from an NIH444 by R265a cross based on read depths from whole genome sequencing. 8 of 14 demonstrate aneuploidy for at least one scaffold. [file elife-28802-fig5-data1.docx]

Figure 5- Figure Supplement 1. Aneuploidy in NIH444 x R265**a** cross

| Spore # | Aneuploid scaffold(s) | *MSH2* allele |
| --- | --- | --- |
| 1 | - | *msh2*del131 |
| 2 | 19,21,26 | WT |
| 3 | - | *msh2*del131 |
| 4 | 5 | *msh2*del131 |
| 5 | 6 | *msh2*del131 |
| 6 | - | *msh2*del131 |
| 7 | 6 | WT |
| 8 | - | WT |
| 9 | 6 | WT |
| 10 | 6 | WT |
| 11 | 6 | WT |
| 12 | - | WT |
| 13 | - | *msh2*del131 |
| 14 | 19,21,26 | *msh2*del131 |
