# Supplementary material for: Natural mismatch repair mutations mediate phenotypic diversity and drug resistance in Cryptococcus deuterogattii
Source: eLife. 2017 Sep 26;6:e28802. doi: 10.7554/eLife.28802 (PMC5614558; doi:10.7554/eLife.28802)
Supplement: Supplementary file 1. [file elife-28802-supp1.docx]

Supplemental File 1: Strains used in this study.

| **Strain** | **Genotype** | **Source/Reference** |
| --- | --- | --- |
| R265 | VGIIa | (32)⁠ |
| EJB17 | VGIIa | (34)⁠ |
| R272 | VGIIb | (33)⁠ |
| NIH444 | VGIIa-like, *msh2*del131 | (33)⁠ |
| NIH444(v) | VGIIa-like, *msh2*del131 | (62)⁠ |
| CBS7750 | VGIIa-like, *msh2*del131 | (33)⁠ |
| ICB107 | VGIIa-like, *msh2*del131 | (33)⁠ |
| RBB17 | R265, *msh2*Δ::*NEO* | This study |
| RBB18 | R265, *msh2*Δ::*NEO* | This study |
| RBB22 | RBB18, *msh2*Δ::*NEO,* *ade2* | This study |
| RBB23 | RBB22, *msh2*Δ::*NEO,* white, *ADE2* | This study |
| RBB24 | RBB22, *msh2*Δ::*NEO,* white, *ade2* | This study |
| RBB25 | RBB22, *msh2*Δ::*NEO,* red, *ade2* | This study |
| RBB26 | RBB22, *msh2*Δ::*NEO,* red, *ade2* | This study |
| ECt1 | XL280 *ade2*::*NEO* | This study |
| R265**a** | R265, *MAT***a** | (40)⁠ |
| SEC016 | NIH444xR265**a** spore #1, *msh2*del131 | This study |
| SEC017 | NIH444xR265**a** spore #2 | This study |
| SEC018 | NIH444xR265**a** spore #3, *MAT***a**, *msh2*del131 | This study |
| SEC019 | NIH444xR265**a** spore #4, *MAT***a**, *msh2*del131 | This study |
| SEC020 | NIH444xR265**a** spore #5, *msh2*del131 | This study |
| SEC021 | NIH444xR265**a** spore #6, *MAT***a**, *msh2*del131 | This study |
| SEC022 | NIH444xR265**a** spore #7 | This study |
| SEC023 | NIH444xR265**a** spore #8 | This study |
| SEC024 | NIH444xR265**a** spore #9, *MAT***a** | This study |
| SEC025 | NIH444xR265**a** spore #10, *MAT***a** | This study |
| SEC026 | NIH444xR265**a** spore #11 | This study |
| SEC027 | NIH444xR265**a** spore #12, *MAT***a** | This study |
| SEC028 | NIH444xR265**a** spore #13, *msh2*del131 | This study |
| SEC029 | NIH444xR265**a** spore #14, *MAT***a**, *msh2*del131 | This study |
| SEC501 | R265, *NEO* | This study |
| SEC559 | SEC016, passaged 4 times at 37°C | This study |
| SEC560 | SEC017, passaged 4 times at 37°C | This study |
| SEC561 | SEC018, passaged 4 times at 37°C | This study |
| SEC562 | SEC019, passaged 4 times at 37°C | This study |
| SEC563 | SEC020, passaged 4 times at 37°C | This study |
| SEC564 | SEC021, passaged 4 times at 37°C | This study |
| SEC565 | SEC022, passaged 4 times at 37°C | This study |
| SEC566 | SEC023, passaged 4 times at 37°C | This study |
| SEC567 | SEC024, passaged 4 times at 37°C | This study |
| SEC568 | SEC025, passaged 4 times at 37°C | This study |
| SEC569 | SEC026, passaged 4 times at 37°C | This study |
| SEC570 | SEC027, passaged 4 times at 37°C | This study |
| SEC571 | SEC028, passaged 4 times at 37°C | This study |
| SEC572 | SEC029, passaged 4 times at 37°C | This study |
| SEC573 | R265**a**, passaged 4 times at 37°C | This study |
| SEC574 | NIH444, passaged 4 times at 37°C | This study |
| SEC575 | SEC562, passaged 5 additional times at 37°C | This study |
