# Supplementary material for: Natural mismatch repair mutations mediate phenotypic diversity and drug resistance in Cryptococcus deuterogattii
Source: eLife. 2017 Sep 26;6:e28802. doi: 10.7554/eLife.28802 (PMC5614558; doi:10.7554/eLife.28802)
Supplement: Supplementary file 2. [file elife-28802-supp2.docx]

Supplemental File 2. Oligonucleotides used in this study.

| **Primer** | **Sequence (5' to 3')** | **Comment** |
| --- | --- | --- |
| M13F | GTAAAACGACGGCCAG | universal oligo |
| M13R | CAGGAAACAGCTATGAC | universal oligo |
| JOHE41021/BB265 | GAGCGTATTTCGAAGCAGG | *MSH2* 5' F deletion |
| JOHE41022/BB266 | CATCTGGCCATAGTGACGC | *MSH2* 5' F nested deletion |
| JOHE41023/BB267 | CTGGCCGTCGTTTTACAAAGACGAACTTCATTATGAGC | *MSH2* 5' R deletion |
| JOHE41024/BB268 | GTCATAGCTGTTTCCTGCCTTGACAAGTTTGATCGC | *MSH2* 3' F deletion |
| JOHE41025/BB269 | CGACATTTGATGAACCTTCACC | *MSH2* 3' R deletion |
| JOHE41026/BB270 | GTCATATACCCGGCACACTTCG | *MSH2* 3' R nested deletion |
| JOHE41027/BB271 | CCGAGAAAGCAGAAGTGACC | *MSH2* F sequencing |
| JOHE41245/BB275 | CCAAGCAGATGCGTATCG | *MSH2* R sequencing |
| JOHE42336/BB280 | CCTTCCAACAGGCCAAAGTGG | *ADE2* 5' F for sequencing |
| JOHE42337/BB281 | GGTAATTTGTGCCTGACTGG | *ADE2* 5' F nested for sequencing |
| JOHE42338/BB282 | GCGAAGTCCAGCCAAGTCC | *ADE2* F ~400 for sequencing |
| JOHE42339/BB283 | GGCTGAGAAGGCAGTCGG | *ADE2* F ~800 for sequencing |
| JOHE42340/BB284 | GGATTGATCTCATTGCACCTCC | *ADE2* F ~1300 for sequencing |
| JOHE42341/BB285 | CCGACATCAAGCCAACAGG | *ADE2* 3' R for sequencing |
| JOHE42342/BB286 | CGTGCTGCAGATGCTGG | *ADE2* 3' R nested for sequencing |
| JOHE42343/BB287 | CCACGCCGTCTAGTACACTGG | *ADE2* R ~1600 for sequencing |
| JOHE42344/BB288 | GCAGCACCAGAAACAGTGAGAGC | *ADE2* R ~1100 for sequencing |
| JOHE42345/BB289 | CCCAACCTTCAGCATAAAGAGG | *ADE2* R ~600 for sequencing |
| JOHE26938/BB14 | GTCTTCCCAAGCCCTCGACTC | *URA5* 5' F for sequencing |
| JOHE26939/BB15 | CCGGTGAGCCATATCGCAGC | *URA5* 5' F nested for sequencing |
| JOHE26941/BB17 | CCTGTACTTCCTGACCTCTCG | *URA5* 3' R for sequencing |
| JOHE26942/BB18 | CCCACTTTCCGGAGCCTTCC | *URA5* 3' R nested for sequencing |
| JOHE40363/BB257 | GTGTTTGGACGAGCAGTCGG | *FRR1* 3' R for sequencing |
| JOHE40364/BB258 | GCAGCAATGCAATCCTGG | *FRR1* 3' R nested for sequencing |
| JOHE40365/BB259 | GGTACAGGGCGTTGGACC | *FRR1* 5' F for sequencing |
| JOHE40366/BB260 | CGACCTGCAATAGTTTCCC | *FRR1* 5' F nested for sequencing |
| JOHE40500/SEC129 | GGGATGACAGGAGATCCTGC | NEO 3' R for insert confirmation |
| JOHE40501/SEC130 | GCAACAATCCATCCGTGCTGG | NEO 5' F for insert confirmation |
