# Supplementary material for: Natural mismatch repair mutations mediate phenotypic diversity and drug resistance in Cryptococcus deuterogattii
Source: eLife. 2017 Sep 26;6:e28802. doi: 10.7554/eLife.28802 (PMC5614558; doi:10.7554/eLife.28802)
Supplement: Transparent reporting form [file elife-28802-transrepform.docx]

***eLife’s* transparent reporting form**

We encourage authors to provide detailed information *within their submission* to facilitate the interpretation and replication of experiments. If you have any questions, please contact us: [editorial@elifesciences.org](mailto:editorial@elifesciences.org).

**Sample-size estimation**

- You should state whether an appropriate sample size was computed when the study was being designed
- You should state the statistical method of sample size computation and any required assumptions
- If no explicit power analysis was used, you should describe how you decided what sample (replicate) size (number) to use

Please outline where this information can be found within the submission (e.g., page numbers or figure legends), or explain why this information doesn’t apply to your submission:

Explicit power analysis was not employed. Throughout we typically sought to maximize replicate size in initial experiments and then test for significance. Using this approach, in this study we did not find ourselves underpowered to test an observed difference between conditions. For example, we have typically used 10 biological replicates as a “rule of thumb” for conducting fluctuation assays. This is easily sufficient to detect changes in mutation rate of the scale observed in this study (and that may be predicted for a null mutation of *msh2*).

**Replicates**

- You should report how often each experiment was performed
- You should include a definition of biological versus technical replication
- The data obtained should be provided and sufficient information should be provided to indicate the number of independent biological and/or technical replicates
- If you encountered any outliers, you should describe how these were handled
- Criteria for exclusion/inclusion of data should be clearly stated
- High-throughput sequence data should be uploaded before submission, with a private link for reviewers provided (these are available from both GEO and ArrayExpress)

Please outline where this information can be found within the submission (e.g., page numbers or figure legends), or explain why this information doesn’t apply to your submission:

We focused on obtaining high numbers of biological replicates throughout the study, and reporting the means of all data where possible (for quantitative data). Individual replicates for fluctuation assays are overnight cultures inoculated from independent single colonies. To test molecular basis of resistance, only a single colony is tested from each overnight culture, to make sampling as random as possible. WGS was performed only a single time (with the exception of NIH444) but replication of genome sequencing is not common in a single study. Semi-quantitative plate swabbing assays to determine mutation rate were performed from a minimum of 4 independent colonies, and up to as many as 48, with representative images shown. Spot dilution assays were performed twice, with similar results in both replicates.

No data was excluded throughout the study, except in Figure 7C, where 1 biological replicate was excluded that yielded only a single colony (NEO competition with FK506/Rapamycin), making estimation of frequency within the competition culture difficult and error prone.

Most of the genome sequence used in this study is already publicly available on the SRA. We have deposited the remaining data under project PRJNA387047 on the SRA.

**Statistical reporting**

- Statistical analysis methods should be described and justified
- Raw data should be presented in figures whenever informative to do so (typically when N per group is less than 10)
- For each experiment, you should identify the statistical tests used, exact values of N, definitions of center, methods of multiple test correction, and dispersion and precision measures (e.g., mean, median, SD, SEM, confidence intervals; and, for the major substantive results, a measure of effect size (e.g., Pearson's r, Cohen's d)
- Report exact p-values wherever possible alongside the summary statistics and 95% confidence intervals. These should be reported for all key questions and not only when the p-value is less than 0.05.

Throughout we utilized students t-tests to determine significance of continuously distributed data, fisher’s exact test for categorical data, chi square for high N categorical data, and the log-rank test for virulence data.

P-values for the relevant comparisons are highlighted in text when the figure is referenced along with the statistical test employed. For example:

Figure 2A: lines 164-165

Figure 2B: lines 172-173

Figure 3B: line 187

Figure 4C: line 223

Figure 4D: line 228

Figure 5G: lines 266-267

Figure 7A: lines 298-299

Figure 8: line 822 (Figure Legend)

Values of N are reported either within figure legends (Figures 2A, 4C, 5G), the text (Figure 3, 180-181), the methods (Figure 7A, line 493), or in the figure itself (Figure 8).

Please outline where this information can be found within the submission (e.g., page numbers or figure legends), or explain why this information doesn’t apply to your submission:

(For large datasets, or papers with a very large number of statistical tests, you may upload a single table file with tests, Ns, etc., with reference to page numbers in the manuscript.)

**Additional data files (“source data”)**

- We encourage you to upload relevant additional data files, such as numerical data that are represented as a graph in a figure, or as a summary table
- Where provided, these should be in the most useful format, and they can be uploaded as “Source data” files linked to a main figure or table
- Include model definition files including the full list of parameters used
- Include code used for data analysis (e.g., R, MatLab)
- Avoid stating that data files are “available upon request”

Please indicate the figures or tables for which source data files have been provided:

Supplemental Figures 1 and 2 have been uploaded as source data for Figures 5 and 6 respectively. Table 1 has been uploaded as source data for Figure 1 and Table 2 has been uploaded as source data for Figure 5. Raw concatenated SNP matrix utilized for maximum parsimony phylogeny uploaded as source data for Figure 8.
